# Association of Sun Exposure, Skin Colour and Body Mass Index with Vitamin D Status in Individuals Who Are Morbidly Obese

**DOI:** 10.3390/nu9101094

**Published:** 2017-10-04

**Authors:** Clare F. Dix, Judith D. Bauer, Ian Martin, Sharon Rochester, Briony Duarte Romero, Johannes B. Prins, Olivia R. L. Wright

**Affiliations:** 1 School of Human Movement and Nutrition Sciences, The University of Queensland, Brisbane, QLD 4076, Australia; j.bauer1@uq.edu.au (J.D.B.); o.wright@uq.edu.au (O.R.L.W.); 2 Wesley Hospital, Auchenflower, Brisbane, QLD 4066, Australia; Ian.Martin@uchealth.com.au (I.M.); smrochester01@gmail.com (S.R.); 3 QIMR Berghofer Medical Research Institute, Brisbane, QLD 4029, Australia; bduarteromero@hotmail.com; 4 Mater Research Institute, South Brisbane, QLD 4101, Australia; john.prins@mater.uq.edu.au

**Keywords:** vitamin D, morbid obesity, sun exposure, skin colour, biomarkers, micronutrients

## Abstract

Vitamin D deficiency is a common issue, particularly in obese populations, and is tested by assessing serum 25(OH)D concentrations. This study aimed to identify factors that contribute to the vitamin D status in fifty morbidly obese individuals recruited prior to bariatric surgery. Data collected included serum 25(OH)D concentrations, dietary and supplement intake of vitamin D, sun exposure measures, skin colour via spectrophotometry, and genotype analysis of several single nucleotide polymorphisms in the vitamin D metabolism pathway. Results showed a significant correlation between serum 25(OH)D concentrations and age, and serum 25(OH)D and ITAC score (natural skin colour). Natural skin colour accounted for 13.5% of variation in serum 25(OH)D, with every 10° increase in ITAC score (i.e., lighter skin) leading to a 9 nmol/L decrease in serum 25(OH)D. Multiple linear regression using age, ITAC score, and average UV index in the three months prior to testing, significantly predicted serum 25(OH)D concentrations (*R*^2^ = 29.7%). Single nucleotide polymorphisms for all vitamin D genes tested, showed lower serum 25(OH)D for those with the rare genotype compared to the common genotype; this was most pronounced for *fok1* and rs4588, where those with the rare genotype were insufficient (<50 nmol/L), and those with the common genotype were sufficient (≥50 nmol/L). Assessing vitamin D status in individuals with morbid obesity requires testing of 25(OH)D, but potential risk factors for this population include natural skin colour and age.

## 1. Introduction

Vitamin D refers to a group of fat-soluble secosteroids that act as a hormone in the body. There are five forms of vitamin D, of which vitamin D_2_ and vitamin D_3_ are physiologically important. Classical physiological roles for vitamin D include calcium homeostasis and bone metabolism [1], but in recent years, a more varied role for vitamin D has been identified [2,3]. The majority of vitamin D_3_ is produced endogenously in the skin from dehydro-cholesterol after exposure to ultraviolet B (UVB) rays. Vitamin D_2_ and vitamin D_3_ are also found in supplements and some food sources. Vitamin D is transported in the blood, attached to a binding protein, and is metabolised in the liver to 25-hydroxyvitamin D (25(OH)D), and in the kidneys to 1α,25-dihydroxyvitamin D (1,25(OH)_2_D). The majority of the active form of vitamin D, 1,25(OH)_2_D, is produced in the kidneys, although almost all tissues in the body have the ability to produce it [4]. 1,25(OH)_2_D has both genomic and non-genomic effects, through either a nuclear or membrane receptor [5,6,7,8].

Assessing an individual’s vitamin D status is a difficult task; currently, serum 25(OH)D concentration is used as a biomarker for vitamin D status. There are many definitions for sufficiency. The Endocrine Society define deficiency as <50 nmol/L (<20 ng/mL), and insufficiency as 52.5–72.5 nmol/L (21–29 ng/mL) [9]. In Australia, serum 25(OH)D concentrations ≥50 nmol/L are considered sufficient for the general population, with graded concentrations of insufficiency; mild (49–30 nmol/L), moderate (29–12.5 nmol/L), and severe (<12.5 nmol/L) [10]. Higher concentrations are recommended for specific sub-groups, e.g., >60 nmol/L for falls prevention in the elderly [11], and >82.5 nmol/L for reducing colorectal cancer risk [2]. In Australia, deficiency affects around 6% of the population in summer and around 49% of the population in winter [12]. In the USA, around 32% of the population are classified as deficient [13]. There are many issues to consider when assessing a persons’ vitamin D status, including age, gender, physical activity levels, sun exposure, skin colour, diet, and supplement intake. The influence of individual genetics of the person assayed may also affect their status. Single nucleotide polymorphisms (SNPs) in the genes that encode for the vitamin D receptor (VDR) [14,15,16] and vitamin D binding protein (DBP) [17,18] have the potential to influence the activity of 1,25(OH)_2_D.

The majority of vitamin D comes from endogenous production that requires exposure of the skin to UVB rays from sunlight. In assessing a person’s vitamin D status, information regarding sun exposure can help identify those at risk of deficiency. Several questionnaires have been developed to assess sun exposure using a combination of questions about clothing, time spent outdoors, sunscreen use and skin colour [19,20]. Those with darker skin colour appear to require long periods of UV exposure to reach sufficient serum 25(OH)D concentrations [21]. Conversely, those with very light skin are also at risk due to increased sun protective behaviours [22]. Previously, measures of natural and tanned skin colour using spectrophotometry have identified associations with vitamin D status [23]. It has been postulated that tanned skin colour is an important determinant of 25(OH)D status [23]. This suggests that the natural skin colour of a person is not as important as the amount of sun exposure they receive when assessing vitamin D status. 

Vitamin D deficiency is very common in obese populations, including bariatric patients [24,25]. There is an inverse correlation between obesity and low vitamin D status, but it is not clear whether vitamin D is a cause or a consequence of obesity. Strengthening the link between the two are the associations between vitamin D deficiency and many of the co-morbidities associated with obesity [26,27,28,29]. There are several theories on the link between vitamin D deficiency and obesity. One of these is volumetric dilution of 25(OH)D through the greater tissue mass of obese individuals, thereby limiting the 25(OH)D in the blood and indicating a lower vitamin D status. Reduced sun exposure, sun protective behaviours and covering of skin could also impact endogenous vitamin D production [24,30]. Both liver and kidney disease are common in obese populations and can impair metabolism of vitamin D to 25(OH)D and then the hormonally-active form—1,25(OH)_2_D [28,31]. Rare alleles for SNPs in the VDR and DBP have been associated with higher body weight and body mass index (BMI), and lower vitamin D status [32,33,34,35], suggesting a potential genetic link between body weight and vitamin D status.

As vitamin D deficiency is an important and common issue for obese individuals, we aimed to investigate the relationship between several factors, including BMI, sun exposure, and skin colour, with vitamin D status in a group of morbidly obese individuals. The aim of this paper is to identify factors beyond the standard 25(OH)D measurement that may aid in assessing vitamin D status of morbidly obese individuals. 

## 2. Materials and Methods

### 2.1. Participants

Participants were recruited as part of a study into vitamin D supplementation post bariatric surgery at the Wesley Hospital, Brisbane, Australia. The data presented here are the pre-surgical information collected. Inclusion criteria included: age ≥18 years, and accepted for bariatric surgery by surgical team. The surgical team use the AACE/TOS/ASMBS Clinical Practice Guidelines for the Perioperative Nutritional, Metabolic, and Nonsurgical Support of the Bariatric Surgery Patient to assess patient suitability for surgery [36]. Exclusion criteria included: pregnancy, age <18 years, taking medications that affected vitamin D levels, vitamin D supplement use in the last three months, or having liver or kidney disease. All subjects gave their informed consent for inclusion before they participated in the study. The study was conducted in accordance with the Declaration of Helsinki, and the protocol was approved by the Human Research Ethics Committee of the University of Queensland (#2015000446) and Uniting Care Human Research Ethics Committee (#1502).

### 2.2. Study Design

In this cross-sectional study, participants were recruited at the time of their initial consultation with the bariatric surgeon. Participant characteristics were collected from study enrolment forms and clinical records. Data collected included pre-surgery 25(OH)D status, age, gender, BMI, season of vitamin D testing, sun exposure behaviours, and assessment of skin colour using spectrophotometry. 

### 2.3. Data Collection

#### 2.3.1. Anthropometry

Weight (kg) and height (m) were measured to the nearest 0.1 kg and 1 cm. Weight and height were measured using digital column scales (SECA 769, Chino, CA, USA). BMI was calculated using weight (kg)/height (m)^2^.

#### 2.3.2. Biochemistry

As part of standard care, the surgical team requested the following biochemical parameters from serum: 25(OH)D, parathyroid hormone, calcium, iron studies (iron, ferritin, transferrin), full blood count (red blood cells, white blood cells, platelets), and liver function tests (aspartate aminotransferase, alkaline phosphatase, alanine aminotransferase, albumin, bilirubin). Participants used one of two pathology laboratories available throughout Queensland. Participants’ biochemistry results were collected, where available, pre-surgery, 3 months, 6 months, and 12 months post-surgery. Parathyroid hormone was measured by immunoassay (Centaur XP; Siemens, Tarrytown, NY, USA or Cobas 8000 E602; Roche Diagnostics, Mannheim, Germany). Calcium was measured by immunoassay (Architect I2000sr; Abbott, Abbott Park, IL, USA or Advia 2400; Siemens, Tarrytown, NY, USA). Iron studies were measured by immunoassay (Architect I2000sr; Abbott, Abbott Park, IL, USA or Advia 2400; Siemens, Tarrytown, NY, USA). Full blood counts were measured by XN-10 Hematology Analyser (Sysmex, Kobe, Japan). Liver function tests were measured using immunoassay (Advia 2400; Siemens, Tarrytown, NY, USA). 

Vitamin D was measured with automated chemiluminescent competitive immunoassay (Liaison XL; DiaSorin, Stillwater, MN, USA or ADVIA Centaur XP; Siemens, Tarrytown, NY, USA). The Liaison XL measurement range is 10–375 nmol/L (4–150 ng/mL). It is reported to demonstrate equimolar cross-reactivity with 25(OH)D_3_ (100%) and 25(OH)D_2_ (104%), and cross reactivity of <1% with 3-epi-25(OH)D_3_ [37]. Precision analysis for Liaison XL have been reported between 12.6 and 10.8% [38]. The Centaur XP measurement range is 10.5–375 nmol/L (4.2–150 ng/mL). It is reported to demonstrate equimolar cross-reactivity with 25(OH)D_3_ (100.7%) and 25(OH)D_2_ (104.5%), and cross-reactivity of 1.1% with 3-epi-25(OH)D_3_ [39]. Precision analysis for Centaur XP have been reported between 4.2 and 11.9% [38]. Both laboratories use the Royal College of Pathologists of Australasia Quality Assurance Program for vitamin D, and one uses the Vitamin D External Quality Assessment Scheme. Vitamin D status was defined using the following ranges: sufficient ≥50 nmol/L, mildly insufficient 49–25 nmol/L, moderately insufficient 24–12.5 nmol/L, and severely insufficient <12.5 nmol/L [10]. 

#### 2.3.3. Sun Exposure

Participants completed a questionnaire on sun exposure and clothing worn on either workdays or non-workdays in the last three months, based on a previously validated survey [40]. From this data, an average sun exposure time per day was calculated (min/day). Using a modified rule of nines method for estimating percentage of Body Surface Area (%BSA) in individuals with obesity [41], an average daily %BSA exposed to the sun was calculated from information on clothing worn each day. 

#### 2.3.4. Skin Colour

Skin colour measurements were taken using a Spectrophotometer CM-2600/D (Konica Minolta, Tokyo, Japan). This instrument measures skin reflectance of light within the wavelength range of 360 nm to 740 nm. The data is reported using the Commission Internationale de L’Eclairage L*a*b* system. Where L* indicates the lightness or brightness of the skin [42]. Readings were taken on the inner arm (natural skin colour) and the outer forearm (tanned skin colour) using the specular component included (SCI) results for L*a*b. Individual typology angles were calculated using the following formula: ITA = (ArcTangent ((L − 50)/b)) × 180/π [43]. Skin colour was then classified using the ITA into the following groups: very light > 55 > light > 41 > intermediate > 28 > tanned > −10 > brown > −30 > dark [43]. ITA calculations were used to create a measure of tan by subtracting the natural skin colour score (ITAC) from the tanned skin colour score (ITAF), i.e., the difference in ITA score between natural and tanned skin.

#### 2.3.5. UV Index

The average UV index in the three months prior to vitamin D testing was recorded for each participant using the data from the Australian Radiation Protection and Nuclear Safety Agency (http://www.arpansa.gov.au). Average UV Index in the three months prior to testing was used as it can take 2–5 months for serum 25(OH)D concentrations to plateau, and it is suggested to not retest for three months [10].

#### 2.3.6. Dietary Vitamin D Intake

Participants completed a diet questionnaire based on a previously validated food frequency questionnaire [44]. Serve sizes were based on the Australia Guide to Healthy Eating [45]. Vitamin D_3_ equivalents per serve were calculated based on the NUTTAB 2011-12 Vitamin D food database [46]. The NUTTAB 2011-12 Vitamin D database determined vitamin D_3_, 25(OH)D_3_, vitamin D_2_, and 25(OH)D_2_ content using normal phase high-performance liquid chromatography, with ultraviolet detection, on an extract of saponified samples of each food. Vitamin D equivalents were calculated with a factor that takes into account the potentially higher bioavailability of the 25-hydroxy forms of vitamin D. Dietary vitamin D equivalents intake for each participant was calculated for foods containing vitamin D, by calculating the vitamin D equivalents per serve, and multiplying by the minimum number of serves per week indicated by the participant’s response (see Table 1).

#### 2.3.7. Single Nucleotide Polymorphisms

DNA was extracted from whole blood samples from 45 participants using QIAamp DNA Blood Mini kit (#51104, Qiagen, Hilden, Germany). Five SNPs were genotyped using a MassARRAY System (Agena Bioscience, San Diego, CA, USA), conducted by the Australian Genomics Research Facility, The University of Queensland, Brisbane, Australia. Participants were identified as either common homozygous, heterozygous, or rare homozygous for each SNP (see Table 2). Global minor allele frequency data was sourced from 1000 Genomes [47].

### 2.4. Statistical Analysis

Statistical analysis was conducted using SPSS 24 (IBM Corp. Released 2015. IBM SPSS Statistics for Macintosh, Version 24.0. Armonk, NY, USA: IBM Corp.). Variables were assessed for normality and transformed where possible. Pearson correlation and Spearman Rank Correlation were used where appropriate. Linear and multiple regression models were used to determine the effect of independent variables on serum 25(OH)D. One-way ANCOVA was used to determine significant differences between mean serum 25(OH)D concentrations while accounting for covariates, and Bonferroni multiple comparisons test was used to identify significant differences between groups. Allelic frequencies were tested against Hardy–Weinburg equilibrium. Significance was set at *p* < 0.05.

## 3. Results

### 3.1. Characteristics

See Table 3 for participant characteristics. Fifty participants were recruited (80% female), the age range was 23 to 61 years, 70% were Obese Class III (>40 kg/m^2^), 58% were vitamin D sufficient (>50 nmol/L), 35% were vitamin D insufficient (<50 nmol/L), and serum 25(OH)D concentrations ranged from 21 to 103 nmol/L with a normal distribution. The majority (83%) had very light/light constitutive skin colour, and 74% had intermediate/tanned facultative skin colour. Sun exposure times ranged from 0 to 309 min/day, and body surface area exposed to the sun ranged from 0 to 52.5%. Skin colour measurements were not conducted on three participants due to equipment malfunction. Average sun exposure time, body surface exposure and dietary vitamin D intake were not reported in all participants, due to missing or inaccurate data reported in the sun and diet questionnaires. 

### 3.2. Correlation Analysis

**Figure 1:**
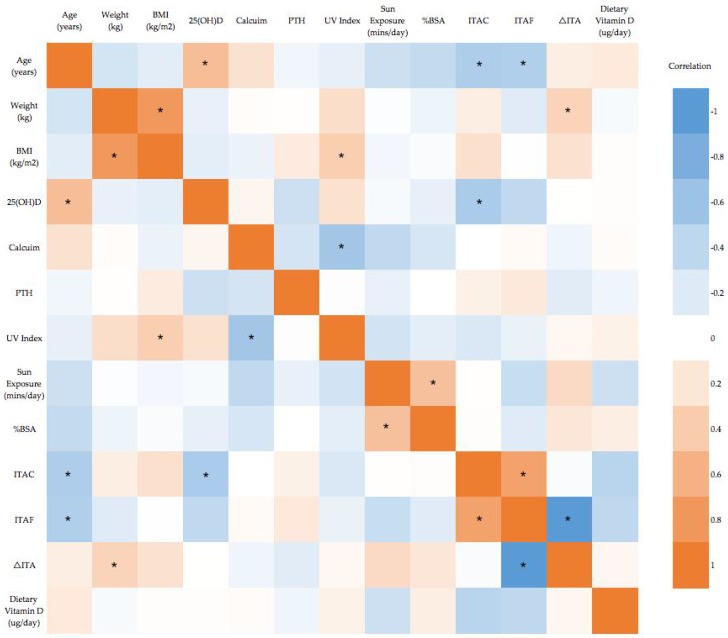
Correlation heat map for all variables. Blue indicates strong negative correlation and Orange indicates strong positive correlation. * *p* < 0.05. BMI body mass index, PTH parathyroid hormone, UV ultraviolet, BSA body surface area, ITA individual typology angle.

Pearson’s or Spearman’s rank correlations were run between all variables (Figure 1). Significant correlations with serum 25(OH)D were found for age and natural skin colour (ITAC). Correlation between serum 25(OH)D and tanned skin colour trended toward significance (*p* = 0.074).

### 3.3. Weight and BMI

No significant correlation was found between weight or BMI, and serum 25(OH)D concentrations. A one-way ANOVA was conducted to determine if serum 25(OH)D concentrations were different between weight or BMI quartiles. There was no significant difference in serum 25(OH)D concentration between weight quartiles, *F*(3, 44) = 0.305, *p* = 0.822, or BMI quartiles, *F*(3, 44) = 1.041, *p* = 0.384 (Table 4).

### 3.4. Dietary Vitamin D

Dietary intake of vitamin D is predicted to contribute 5–10% of total vitamin D intake in Australian populations [10]. The Recommended Daily Intakes for vitamin D are 5 μg/day for those aged 19–50 years, and 10 μg for those aged 51–70 years. Average maximum dietary intake prior to surgery was calculated as 1.9 μg/day for this group of individuals, with a range of 0 to 4.7 μg/day. The average Australia adult is estimated to obtain 1.2 to 2.6 μg/day [48] showing this population is within the normal range for Australian adults. There was no significant correlation between dietary vitamin D and serum 25(OH)D concentrations (Figure 1).

### 3.5. Skin Colour

There was a significant correlation between natural skin colour (ITAC) and serum 25(OH)D concentrations, a trend towards a significant correlation between tanned skin colour (ITAC) (*p* = 0.074) and serum 25(OH)D concentrations, and no significant correlation between degree of tan (ΔITA) and serum 25(OH)D concentrations (Figure 1). ITAC was significantly correlated with age, (*r* = −0.315, *p* = 0.015) and ITAF (*r* = 0.716, *p* < 0.01). ITAF was significantly correlated with age (*r* = −0.340, *p* = 0.019), and change in ITA (*r* = 0.719, *p* < 0.01).

Linear regression analysis was completed to assess the relationship between natural skin colour (ITAC) and serum 25(OH)D concentrations. Natural skin colour accounted for 13.5% of the variation in serum 25(OH)D concentrations, (adj. *R*^2^ = 11%). Natural skin colour significantly predicted serum 25(OH)D concentrations, *F*(1, 43) = 6.711, *p* = .013. For each 10° increase of ITAC score (i.e., lighter natural skin colour), serum 25(OH)D concentrations decrease by 9 nmol/L. Serum 25(OH)D concentrations were predicted using this model for each skin colour group (Table 5). 

Linear regression analysis was completed to assess the relationship between tanned skin colour (ITAF) and serum 25(OH)D concentrations. Tanned skin colour accounted for 7.2% of the variation in serum 25(OH)D concentrations, (adj. *R*^2^ = 5.1%). Tanned skin colour was trending toward significance with serum 25(OH)D concentrations, *F*(1, 43) = 3.344, *p* = 0.074. For each 10° decrease in ITAF score (i.e., darker tan) serum 25(OH)D concentrations increase by 5 nmol/L. Serum 25(OH)D concentrations were predicted using this model for each skin colour group (Table 6).

### 3.6. Sun Exposure

Sun exposure was measured through the sun exposure questionnaire, where participants reported the times spent in direct sunlight, and clothing worn at that time. From this information, an average minutes of sun exposure per day and the %BSA exposed to the sun were calculated. There was no correlation between %BSA or sun exposure minutes and serum 25(OH)D concentrations (Figure 1). Sun exposure minutes and %BSA were significantly positively correlated (*r* = 0.486, *p* = 0.001), suggesting those who spent more time in the sun had more skin exposed. 

### 3.7. Single Nucleotide Polymorphisms

No statistically significant differences in mean serum 25(OH)D concentration were found for any SNP genotype (Table 7); however, lower serum 25(OH)D concentrations were found in the rare genotype compared to the common genotype for all vitamin D SNPs (*bsm1*, *taq1*, *fok1*, rs4588, rs7041). Combinations of two rare genotypes were also examined for any differences in serum 25(OH)D concentrations, weight, or BMI. No significant differences in serum 25(OH)D, weight or BMI were found between those with or without both rare genotypes for *bsm1* and *taq1* (*n* = 8), or rs4588 and rs7041 (*n* = 14), or *fok1* and rs4588 (*n* = 10).

### 3.8. Multiple Regression Model

A multiple regression model was developed to identify the contributions of independent variables to serum 25(OH)D concentrations. Variables were chosen based on their univariate relationship with serum 25(OH)D concentrations, and effect sizes ≥ intermediate. Independent variables included in the model were age, ITAC, and UV Index. The multiple regression model statistically significantly predicted serum 25(OH)D concentrations, *F*(3, 41) = 7.202, *p* = 0.001, adj. *R*^2^ = 29.7%, intermediate effect size [49] (Table 8). Age was the only variable that significantly predicted serum 25(OH)D, showing each year increase in age is associated with an increase of 0.9 nmol/L serum 25(OH)D.

## 4. Discussion

Determinants of vitamin D status in morbidly obese individuals were examined, and factors considered were dietary vitamin D intake, BMI, skin colour, and sun exposure. The key findings were (i) natural skin colour accounted for 13.5% of the variation in serum 25(OH)D concentrations; (ii) there was a significant positive association between age and serum 25(OH)D; (iii) weight and BMI were not significantly associated with serum 25(OH)D concentrations; (iv) there was no relationship between sun exposure time, or amount of skin exposed, with 25(OH)D concentrations in this group. 

Natural skin colour (ITAC score) accounted for 13.5% of the variation in serum 25(OH)D concentrations. Results showed that as natural skin colour becomes darker, serum 25(OH)D concentrations increased, suggesting those with darker natural skin colour have higher 25(OH)D concentrations. ITAC score was also used to predict maximal mean serum 25(OH)D concentrations for each skin colour category. It is well documented that those with darker natural skin colour have lower serum 25(OH)D concentrations [21,50]. As this study had only recruited participants with an intermediate skin colour or lighter, it is possible the increase in 25(OH)D concentration with increasing natural skin colour was due to sun protective behaviours from those with lighter skin. For similar reasons, the predictive model became unreliable when dealing with those in the tanned and brown skin colour categories. Although when comparing changes in ITA score i.e., degree of tanning, sun exposure times, and %BSA, there was no correlation between any of these measures and natural skin colour, potentially suggesting no differences in sun protective behaviours regardless of natural skin colour.

A trend towards significance was seen between tanned skin colour (ITAF score) and serum 25(OH)D concentrations. Tanned skin colour accounted for 7.2% of the variation in serum 25(OH)D concentrations in this group. As ITAF score increased (i.e., darker tan), 25(OH)D concentrations increased, suggesting that those with a darker tan (up to intermediate) will have higher 25(OH)D concentrations. Previous research has shown that tanned skin colour is a significant predictor of 25(OH)D [23]. This result came from a larger group but with similar proportions of participants in each skin colour group. Our results confirm those of Rockell et al. [23], showing that for each 10° decrease in ITAF score (i.e., darker tan) serum 25(OH)D concentrations increase by 5 nmol/L [23].

The Australian Health Survey (2011–12) showed similar rates of deficiency between genders, and an increase in vitamin D status with age, concurrent with increases in supplement use [12]. Population data in Australia show varying effects of age on serum 25(OH)D, with some showing an increase with age [12,51] and others a decrease with age [19]. There was a significant positive association between age and serum 25(OH)D concentrations in our study. Further investigation of the variables showed that age positively correlated with natural skin colour and tanned skin colour, suggesting that the older participants had darker natural and tanned skin colour. There was no relationship between age and degree of tan, or %BSA exposed to the sun, or sun exposure time.

Weight and BMI were not significantly associated with serum 25(OH)D concentrations. There are several meta-analyses that have shown a significant decrease in serum 25(OH)D concentrations with increasing weight or BMI, although not all included morbidly obese individuals [52,53,54]. There are several studies in morbidly obese individuals that found a significant [55,56,57,58,59,60,61] or borderline significant [62,63] inverse relationship between BMI and 25(OH)D concentrations, with small to large effect sizes. It is possible that once a certain BMI threshold is reached, the dilution effects of obesity on vitamin D status plateau, and the effect becomes minimal, hence the trend of lower serum 25(OH)D in this study, but the lack of a significant difference. Sun exposure, or lack of it, appears to have a major influence on vitamin D status in Australian obese populations. A study into determinants of serum 25(OH)D in Australian Adults reported that the amount of skin exposed to the sun was the single largest contributor to serum 25(OH)D concentrations, followed by location, season, and personal UV radiation exposure [19]. BMI only explained 4% of the variance in their population, which include a wide range of BMI [19].

As the majority of vitamin D_3_ is produced endogenously in the skin, it is logical to expect that a relationship would exist between sun exposure times, the %BSA exposed, and vitamin D status. There was no relationship between sun exposure times or %BSA, with serum 25(OH)D concentrations in our study. Previous research into determinants of vitamin D status in Australian adults found a significant association between vitamin D status and time spent outdoors (*r_s_* = 0.16, *p* < 0.0001), and vitamin D status and the percentage of clothing cover (*r_s_* = −0.50, *p* < 0.001), in a population with a wide range of BMI, including individuals with obesity [19]. There are a few possible reasons why no relationship was identified between these measures and vitamin D status in our study. This study reports behaviours of a specific group of morbidly obese individuals, and so represented only their sun exposure practices, whereas the AusD study included a wide range of BMI and a larger sample size. The data provided on sun exposure times and body surface area exposed was self-reported data, and may not have been accurate. There was a trend toward a positive correlation between change in ITA (degree of tan) and time in the sun. This would be expected, as longer sun exposure would generally lead to an increase in tan, providing some evidence that this measure of sun exposure is accurate. Vitamin D is stored in adipose tissue [64], and there is some evidence that muscle may store 25(OH)D [65], thereby reducing the amount of 25(OH)D available in the circulation for measurement. This may impact the ability to correlate vitamin D status with any measures of skin colour, or sun exposure, especially in those with high amounts of adipose tissue or large muscle mass, as seen in this group of morbidly obese individuals. 

Three SNPs for the VDR and two for the DBP were analysed in this group. No significant differences in serum 25(OH)D concentrations were seen between genotypes of any gene. Although all homozygous rare genotypes had lower mean serum 25(OH)D concentrations than the common homozygous genotype. From a clinical perspective, VDR *fok1* and DBP rs4588 both indicated insufficient vitamin D status for those with homozygous rare genotypes compared to homozygous common genotypes. Rare alleles for all four of the main variants for the VDR gene have been associated with lower serum 25(OH)D concentration and vitamin D deficiency in a range of populations [15,16,17,66,67,68]. This suggests a possible method of personalised nutrition in this population, by considering specialised treatments for the patients with a genetic predisposition for lower serum 25(OH)D, based on the SNP genotype, particularly for *fok1* and rs4588. This information could contribute to the overall assessment of vitamin D status, and identify those that may achieve more benefit from supplementation. 

The VDR *bsm1* rare allele has been associated with higher weight, BMI [32,33], and lower percentage of excess weight loss in bariatric patients [69]. The *taq1* rare allele has also been associated with higher BMI and weight [34]. The *fok1* rare allele has not been associated with weight and BMI previously [70,71,72]. In this group of morbidly obese individuals, there was no statistically significant difference in weight, BMI or %EWL, between rare or common genotypes for *bsm1*, *taq1*, and *fok1*. Of clinical significance was the difference in mean serum 25(OH)D concentrations between common homozygous and rare homozygous individuals for *fok1* and rs4588, where the common homozygous genotype was vitamin D sufficient, and the rare homozygous genotype was vitamin D deficient. 

Both DBP SNP common alleles have been associated with higher BMI in females, but not males [35]. We found no significant differences in weight or BMI between the genotypes for both DBP SNPs. The difference in results could be related to differences in sample populations, this study only included patients with BMI > 30, whereas the previous study included a wide range of BMI (16.93–57.21 kg/m^2^) [35]. It is important to note that the SNP analysis from this study was under powered, and so results must be considered with caution. Particularly those that conflict with results from adequately powered studies. Post hoc power analysis using G*Power [73] indicated that power ranged from 6 to 63% for these analyses, and required sample sizes from 69 to 957 for 80% power. 

There are several limitations to this study. Serum 25(OH)D concentrations were measured using chemiluminescent competitive immunoassays, on two different platforms. The gold standard in measuring 25(OH)D concentration is liquid chromatography with tandem mass spectrometry. Issues with measuring 25(OH)D arise from the ability of these assays to release 25(OH)D from its binding protein or other carriers like albumin, the hydrophobic properties of 25(OH)D, and the differing antibody specificities to the metabolites [9]. Although both pathology laboratories reported using quality assurance programs, both platforms have potential issues with under or over recovering metabolites, negative biases, and large deviations at lower concentrations of 25(OH)D (~50 nmo/L) [38]. This highlights an issue for clinicians in interpreting results for the 25(OH)D assay. Recruitment did not occur over a full year, this led to lower numbers of participants recruited post summer and autumn. This influences the results of differences in serum 25(OH)D between seasons, and potentially, the range of tanned skin colours. Using a small range of BMI has also minimised the effects of weight and BMI on serum 25(OH)D; use of a wider range, including normal, overweight, and obese individuals may have shown a more pronounced effect. A similar issue is found with the skin colour assessments, as individuals recruited only had natural skin colour from very light to intermediate. This limits the ability of results to be applied to those with darker natural skin colour. Our sample size was also under powered for SNP analysis, and so results should be considered with caution.

## 5. Conclusions

In this group of morbidly obese individuals lighter natural skin colour and younger age are potential risk factors for vitamin D insufficiency. It appears sun exposure time, the amount of skin exposed to the sun, weight and BMI, do not influence vitamin D status in this population. The vitamin D pathway SNPs investigated showed no statistically significant effects on vitamin D status, although clinically significant differences were found for VDR *fok1* and DBP rs4588. Future research into the determinants of vitamin D status using a larger group of morbidly obese individuals could provide further information on the genetic susceptibility to vitamin D deficiency in this at-risk group. A group with a wider range of natural skin colours could also help to develop an understanding of the contribution of skin colour and tanned skin colour to vitamin D status.

## Figures and Tables

**Table 1:** Vitamin D equivalents calculations by response option.

Vitamin D Source	Serve Size	0–1 Serves Per Week (μg/week)	1 to 4 Serves Per Week (μg/week)	5+ Serves Per Week (μg/week)
Beef	65 g cooked	0.0	0.3	1.3
Canned fish, tuna	85 g	0.0	2.0	10.2
Mushrooms	75 g	0.0	1.7	8.6
Eggs, whole	120 g	0.0	2.5	12.6
Milk, whole	250 mL	0.0	0.3	1.5
Salmon	100 g	0.0	20.0	100.0
Diary blend spread	10 g	0.0	1.0	5.0

Based on source: NUTTAB 2010 (Food Standards Australia New Zealand); The University of New South Wales; Professor Heather Greenfield and co-workers at the University of New South Wales; Tables of composition of Australian Aboriginal Foods (J Brand-Miller, KW James and PMA Maggiore).

**Table 2:** Single nucleotide polymorphisms of interest.

SNP	Gene	MAF	Common Homozygous	Heterozygous	Rare Homozygous
Rs1544410 (*bsm1*)	VDR	0.2959	GG	GA	AA
Rs2228570 (*fok1*)	VDR	0.3285	CC	TC	TT
Rs731236 (*taq1*)	VDR	0.2766	TT	TC	CC
Rs4588	DBP	0.2079	CC	CA	AA
Rs7041	DBP	0.3816	GG	GT	TT

MAF minor allele frequency.

**Table 3:** Participant characteristics.

	*n*	Mean (SD)	95% CI
Weight (kg)	50	126.7 (24.4)	119.8–133.7
BMI (kg/m^2^)	50	43.9 (7.3)	41.8–46.0
Plasma 25(OH)D (nmol/L)	48	56.8 (20.3)	50.9–62.7
Natural skin colour (ITAC score)	47	50.2 (8.2)	47.8–52.6
Tanned skin colour (ITAF score)	47	26.6 (11.7)	23.1–30.0
Degree of tan (ITAC–ITAF)	47	23.6 (8.2)	21.2–26.0
Average sun exposure (min/day)	42	65.4 (64.9)	45.2–85.7
Average BSA exposed (%)	41	17.6 (14.9)	12.9–22.3
Dietary Vitamin D (ug/day)	40	1.9 (1.4)	1.4–2.4

BMI body mass index, BSA body surface area, 25(OH)D 25-hydroxyvitamin D, SD standard deviation.

**Table 4:** Serum 25(OH)D concentrations by weight and BMI quartile. Serum 25(OH)D is presented as mean ± standard deviation.

Quartile	Weight (kg)	Serum 25(OH)D (nmol/L)	BMI (kg/m^2^)	Serum 25(OH)D (nmol/L)
1	≤107 kg	59.5 ± 21.5	≤39	58.9 ± 22.4
2	108–123 kg	59.8 ± 18.3	40–42.6	64.1 ± 22.3
3	124–141 kg	54.2 ± 23.7	42.7–46.8	50.5 ± 18.2
4	≥142 kg	53.7 ± 18.1	≥42.6	53.8 ± 17.5

**Table 5:** Predicted serum 25(OH)D concentrations from linear regression model for ITAC score.

ITAC Score	Serum 25(OH)D Range (nmol/L)
Very light	<53
Light	54–66
Intermediate	67–78
Tanned	79–95
Brown	96–132
Dark	>133

**Table 6:** Predicted serum 25(OH)D concentrations from linear regression model for ITAF score.

ITAF Score	Serum 25(OH)D Range (nmol/L)
Very light	<44
Light	45–51
Intermediate	52–57
Tanned	58–66
Brown	67–85
Dark	>85

**Table 7:** Minor allele frequencies for each SNP, and mean serum 25(OH)D concentration, weight, and BMI by genotype for SNPs of interest. *p* values calculated from one-way ANOVAs.

SNP	MAF	Genotype	*n*	Serum 25(OH)D (nmol/L)	*p* Value	Weight (kg)	*p* Value	BMI (kg/m^2^)	*p* Value
Rs1544410 (*bsm1*)		GG	18	57.8 (22.9)	0.820	118.4 (23.9)	0.340	42.5 (7.5)	0.426 ^a^
		GA	17	54.2 (17.1)		130.7 (27.8)		45.5 (8.8)	
	0.38	AA ^b^	8	53.4 (19.0)		126.0 (18.1)		44.9 (6.2)	
Rs2228570 (*fok1*)		CC	18	59.5 (20.6)	0.238	123.4 (31.5)	0.585 ^a^	44.0 (9.7)	0.342 ^a^
		CT	19	55.6 (20.1)		124.1 (18.4)		43.7 (6.8)	
	0.36	TT ^b^	6	43.7 (11.1)		130.5 (22.7)		45.8 (3.6)	
Rs731236 (*taq1*)		TT	19	57.3 (22.4)	0.878	117.7 (23.4)	0.213	42.2 (7.4)	0.238 ^a^
		TC	16	54.7 (17.6)		132.4 (27.8)		46.0 (8.7)	
	0.37	CC ^b^	8	53.4 (19.0)		126.0 (18.1)		44.9 (6.2)	
Rs4588		CC	23	56.9 (20.4)	0.210	121.0 (25.7)	0.117 ^a^	44.0 (7.5)	0.440 ^a^
		CA	16	57.8 (19.0)		125.4 (24.8)		42.9 (7.7)	
	0.28	AA ^b^	4	39.0 (12.7)		142.7 (8.7)		49.4 (9.8)	
Rs7041		GG	14	59.6 (20.9)	0.601	123.4 (31.6)	0.275 ^a^	45.7 (8.7)	0.237 ^a^
		GT	23	54.4 (19.1)		122.6 (21.1)		42.0 (6.6)	
	0.41	TT ^b^	6	50.7 (20.6)		135.5 (20.5)		48.3 (8.5)	

^a^ Kruskal–Wallis H test; ^b^ homozygous risk genotype, SNP single nucleotide polymorphism, MAF minor allele frequency, BMI body mass index.

**Table 8:** Multiple regression coefficients.

Model	B	SE_B_	β	*p* Value
Constant	19.588	28.908		0.502
Age	0.932	0.273	0.467	0.001*
ITAC	−0.444	0.346	−0.176	0.206
UV Index	3.528	1.965	0.232	0.080

* *p* < 0.05, B unstandardized coefficient, SE_B_ standard error of unstandardized coefficient, β beta, ITA individual typology angle, UV ultraviolet.
